# Laparoscopic diverticulectomy and ureteric reimplantation for congenital bladder diverticulum

**DOI:** 10.1093/jscr/rjag637

**Published:** 2026-07-29

**Authors:** Christos Costa, Arindam Dastidar, Ashok Rijhwani, Subhasis Chakraborty, Foteini Moniati, Kristina Dzhuma

**Affiliations:** Department of Paediatric Urology, John Radcliffe Hospital, Oxford University Hospitals NHS Foundation Trust, Oxford, United Kingdom; Department of Paediatric Urology, John Radcliffe Hospital, Oxford University Hospitals NHS Foundation Trust, Oxford, United Kingdom; Department of Paediatric Urology, John Radcliffe Hospital, Oxford University Hospitals NHS Foundation Trust, Oxford, United Kingdom; Department of Paediatric Radiology, John Radcliffe Hospital, Oxford University Hospitals NHS Foundation Trust, Oxford, United Kingdom; Department of Inflammation and Ageing, University of Birmingham, Birmingham, United Kingdom; Department of Paediatric Urology, John Radcliffe Hospital, Oxford University Hospitals NHS Foundation Trust, Oxford, United Kingdom

**Keywords:** bladder diverticulum, ureteric reimplantation, paediatric urology, laparoscopy, vesicoureteric reflux, urosepsis

## Abstract

Congenital bladder diverticula are rare anomalies that may be associated with vesicoureteric reflux, ureteric ectopia, and impaired bladder emptying. We report a male infant initially found to have distal ureteric dilatation on postnatal ultrasonography. Micturating cystourethrogram demonstrated a contrast-filled structure adjacent to the bladder, raising suspicion of distal ureteric dilatation or bladder diverticulum. Serial imaging confirmed persistence of the lesion. By 3 years of age, the patient developed recurrent urosepsis and voiding dysfunction. Further assessment demonstrated a large bladder diverticulum with ureteric insertion. The patient underwent cystoscopy and laparoscopic diverticulectomy with ureteric reimplantation. Intraoperative findings confirmed a large diverticulum with ureteric insertion into its mid-portion. Reconstruction was achieved without complication, with resolution of symptoms and improved bladder emptying at 3 months follow-up. This case highlights the diagnostic challenges of distal ureteric dilatation and demonstrates the role of minimally invasive surgery in managing complex lower urinary tract anomalies.

## Introduction

Congenital bladder diverticula are uncommon anomalies resulting from focal detrusor weakness, leading to mucosal herniation through the bladder wall. They may occur in isolation or with vesicoureteric reflux or ureteric abnormalities [1, 2]. While many diverticula remain asymptomatic, larger lesions may become clinically significant due to urinary stasis, recurrent infection, and impaired bladder emptying.

Diagnosis can be challenging, particularly in infancy, where imaging may mimic distal ureteric dilatation or other cystic pelvic structures [3]. Differentiation is important as management strategies differ and may require reconstructive surgery. Early recognition and longitudinal imaging are often necessary to clarify anatomy and guide management.

We present a case of a congenital bladder diverticulum with ureteric insertion, requiring surgical reconstruction due to recurrent urosepsis and bladder dysfunction.

## Case report

A male infant born in July 2022 following an uncomplicated pregnancy with normal antenatal imaging was referred at 7 weeks after incidental ultrasonographic findings (US for congenital asplenia in family) of distal left ureteric dilatation (16 mm) with mild renal pelvic dilatation (5.5 mm). The right kidney was normal. Colour Doppler suggested retrograde flow at the vesicoureteric junction, raising suspicion for vesicoureteric reflux, and trimethoprim prophylaxis was commenced.

**Figure 1:**
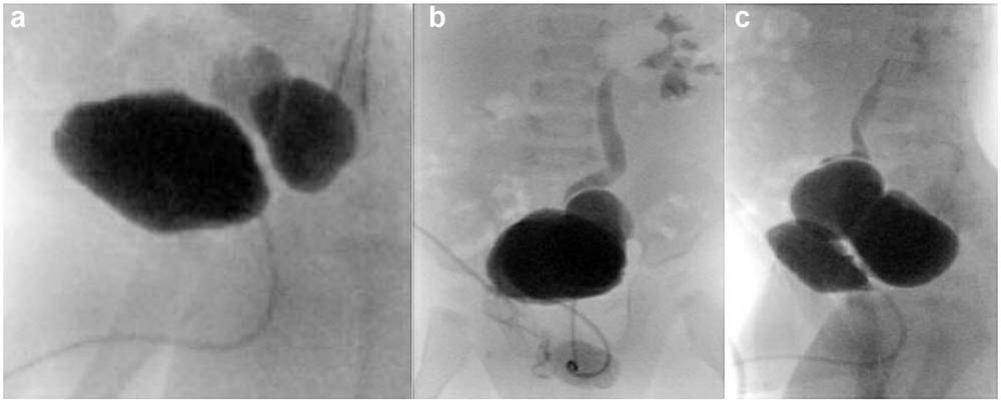
Micturating cystourethrogram demonstrating initial normal bladder filling (a), followed by identification of a contrast-filled structure located to the left and posterior to the bladder (a). The structure communicates with the bladder and enlarges during voiding (b). During the voiding phase, a ureter is visualized arising from the superior aspect of this structure, with contrast reflux extending to the left kidney (b, c). No vesicoureteric reflux is observed on the right side. These findings are indeterminate, with differential considerations including focal dilatation of the distal left ureter and a large bladder diverticulum with ureteric insertion.

**Figure 2:**
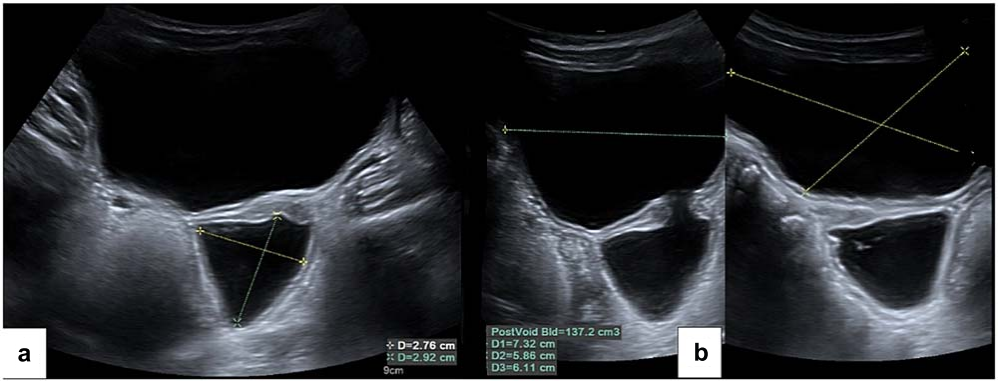
Ultrasound of the urinary tract demonstrating a thin-walled urinary bladder with a pre-void volume of 272 ml (a) and a significant post-void residual volume of 137 ml (b). A cystic structure consistent with a bladder diverticulum is identified arising from the left posteroinferior aspect of the bladder, measuring 29 × 27 mm, with associated urothelial thickening. Colour Doppler demonstrates urinary jets passing from the diverticulum into the bladder lumen.

The case was discussed at a paediatric urology multidisciplinary meeting, and further evaluation was recommended. Micturating cystourethrogram demonstrated a contrast-filled structure posterior to the bladder enlarging during voiding, with reflux extending to the kidney. A ureter appeared to arise from the superior aspect of this structure. Findings were indeterminate between distal ureteric dilatation and a bladder diverticulum with ureteric insertion (Fig. 1a–c). Follow-up US imaging demonstrated persistence of a cystic structure communicating with the bladder via a narrow neck, increasingly suggestive of a diverticulum, with stable renal appearances (Fig. 2a and b). The patient remained clinically well initially. However, by 2.5 years of age, he developed recurrent urosepsis despite prophylaxis, prompting a change to nitrofurantoin. By 3 years, he exhibited urinary hesitancy, intermittent voiding, and incomplete bladder emptying. Bladder function assessment demonstrated increased bladder capacity (>200 ml), staccato flow, and significant post-void residual (~70 ml), consistent with impaired bladder emptying.

Given recurrent infections and functional deterioration in the context of persistent anatomical abnormality, surgical intervention was undertaken.

**Figure 3:**
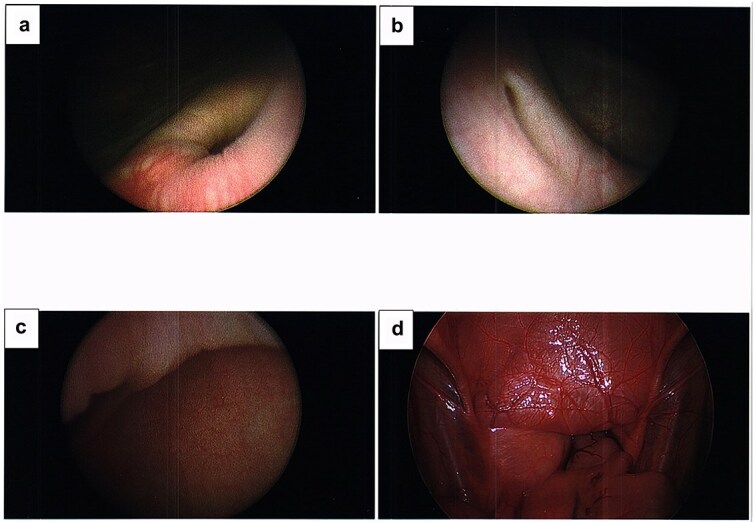
Cystoscopic images demonstrating the bladder diverticulum. (a) The opening of the diverticulum within the left posterolateral bladder wall. (b) The narrow neck of the diverticulum viewed from within the bladder. (c) The interior of the diverticulum cavity showing smooth mucosal lining. (d) Detailed view of the mucosal surface within the diverticulum.

Cystoscopy demonstrated a normal urethra, bladder neck, and orthotopic right ureteric orifice. A large diverticulum arising from the left posterolateral bladder wall with a narrow neck was identified (Fig. 3a–d). The left ureteric orifice was not visualized within the native bladder, supporting ureteric insertion into the diverticulum.

The patient was repositioned supine for the laparoscopic phase. A transumbilical camera port was inserted using an open technique and pneumoperitoneum established at 8 mmHg, with additional working ports placed under direct vision. Laparoscopy confirmed a large diverticulum with the left ureter inserting into its mid-portion. The peritoneum overlying the diverticulum was incised, and meticulous dissection was undertaken to mobilize both the diverticulum and ureter, using a combination of harmonic scalpel and monopolar diathermy to develop safe tissue planes.

The ureter was carefully mobilized distally and divided at its insertion into the diverticulum, preserving peri-ureteric adventitia to maintain vascular supply. A hitch stitch was applied to optimize exposure within the deep pelvis. The diverticulum was mobilized circumferentially to its neck, ligated with an endoloop, and excised. The detrusor defect was closed with interrupted 3/0 Vicryl sutures, and bladder integrity was confirmed with saline distension.

Attention was then turned to ureteric reimplantation. The bladder was hitched to improve exposure, and a detrusor tunnel was created. The ureter was spatulated and reimplanted using a Shanfield-type technique. A 4.7 Fr JJ stent was inserted over a guidewire, and the ureter was secured with interrupted sutures. The detrusor was closed over the ureter to create an antireflux mechanism.

Haemostasis was confirmed and ports removed under direct vision. The operative steps are demonstrated in the supplementary video (Video 1). Histopathological analysis of the excised specimen demonstrated a cystic structure lined by transitional epithelium with haphazard smooth muscle arrangement and focal epithelial hyperplasia. No inflammation or morphological evidence of malignancy was identified, consistent with a congenital bladder diverticulum.

The postoperative course was uncomplicated, with discharge on postoperative Day 2. The urethral catheter was removed at 2 weeks and the JJ stent at 8 weeks, both without complication. At 3 months postoperatively, the patient remains well with no urinary tract infections. Follow-up ultrasonography confirmed surgical absence of the diverticulum with improved bladder emptying (post-void residual 22 ml). Nitrofurantoin prophylaxis is ongoing with planned discontinuation following further review.

## Discussion

Congenital bladder diverticula are rare but clinically significant when associated with infection, urinary stasis, or bladder dysfunction [1]. This case highlights the diagnostic difficulty in distinguishing diverticula from distal ureteric dilatation, particularly in infancy where anatomical relationships may be unclear [3]. Serial imaging demonstrating a persistent cystic structure with a narrow communication to the bladder was key in establishing the diagnosis.

This underscores the limitations of single-modality imaging in infancy, where a combination of ultrasonography and micturating cystourethrogram, interpreted longitudinally, is essential for diagnosis.

Management depends on symptoms. While asymptomatic diverticula may be observed, surgical intervention is indicated in cases of recurrent infection, reflux, or bladder dysfunction [1, 4]. In this patient, progression to urosepsis and voiding dysfunction necessitated intervention.

Ureteric insertion into the diverticulum increases complexity and requires reconstruction to restore effective drainage and prevent reflux [2]. Key surgical principles include complete excision of the diverticulum, preservation of ureteric vascularity, and creation of an adequate detrusor tunnel to ensure an antireflux mechanism [5].

Achieving a tension-free anastomosis with adequate tunnel length to diameter ratio is critical in preventing reflux and obstruction.

The laparoscopic approach provides excellent visualization of pelvic anatomy and facilitates precise dissection and reconstruction. Minimally invasive techniques have demonstrated comparable outcomes to open surgery with reduced morbidity in paediatric urology [6].

This case emphasizes the importance of accurate diagnosis, appropriate timing of intervention, and tailored surgical management in complex lower urinary tract anomalies.

### Learning points

- Congenital bladder diverticula may mimic distal ureteric dilatation on initial imaging; serial assessment with ultrasonography and micturating cystourethrogram is essential for accurate diagnosis.
- Ureteric insertion into the diverticulum necessitates combined diverticulectomy and reimplantation to restore effective drainage and establish an antireflux mechanism.
- Symptomatic diverticula with recurrent infection or voiding dysfunction warrant surgical intervention, with timing guided by clinical progression.
- Laparoscopic excision provides excellent pelvic visualization and enables precise reconstruction in paediatric patients.

## Supplementary Material

bladder_diverticulum_small_version_rjag637

Supplementary_Video_1_caption_rjag637
