# Supplementary material for: Laparoscopic diverticulectomy and ureteric reimplantation for congenital bladder diverticulum
Source: J Surg Case Rep. 2026 Jul 29;2026(7):rjag637. doi: 10.1093/jscr/rjag637 (PMC13418609; doi:10.1093/jscr/rjag637)
Supplement: Supplementary_Video_1_caption_rjag637 [file supplementary_video_1_caption_rjag637.docx]

**Video 1:** Supplementary video demonstrating the operative steps of cystoscopy-guided laparoscopic diverticulectomy and ureteric reimplantation.
